# Challenges and Pitfalls of Research Designs Involving Magnesium-Based Biomaterials: An Overview

**DOI:** 10.3390/ijms25116242

**Published:** 2024-06-05

**Authors:** Nourhan Hassan, Thomas Krieg, Alexander Kopp, Alexander D. Bach, Nadja Kröger

**Affiliations:** 1 Department of Plastic, Reconstructive and Aesthetic Surgery, University Hospital Cologne, 50937 Cologne, Germany; 2 Institute for Laboratory Animal Science and Experimental Surgery, University of Aachen Medical Center, Faculty of Medicine, RWTH-Aachen University, 52074 Aachen, Germany; 3 Biotechnology Department, Faculty of Science, Cairo University, Giza 12613, Egypt; 4 Translational Matrix Biology, Medical Faculty, University of Cologne, 50937 Cologne, Germany; 5 Cologne Excellence Cluster on Cellular Stress Responses in Aging-Associated Diseases (CECAD), University of Cologne, 50937 Cologne, Germany; 6 Center for Molecular Medicine (CMMC), University of Cologne, 50937 Cologne, Germany; 7 Meotec GmbH, Philipsstr. 8, 52068 Aachen, Germany; 8 Department of Plastic, Aesthetic and Hand Surgery, St. Antonius Hospital Eschweiler, 52249 Eschweiler, Germany

**Keywords:** magnesium, implant, animal model, in vitro, biomaterial

## Abstract

Magnesium-based biomaterials hold remarkable promise for various clinical applications, offering advantages such as reduced stress-shielding and enhanced bone strengthening and vascular remodeling compared to traditional materials. However, ensuring the quality of preclinical research is crucial for the development of these implants. To achieve implant success, an understanding of the cellular responses post-implantation, proper model selection, and good study design are crucial. There are several challenges to reaching a safe and effective translation of laboratory findings into clinical practice. The utilization of Mg-based biomedical devices eliminates the need for biomaterial removal surgery post-healing and mitigates adverse effects associated with permanent biomaterial implantation. However, the high corrosion rate of Mg-based implants poses challenges such as unexpected degradation, structural failure, hydrogen evolution, alkalization, and cytotoxicity. The biocompatibility and degradability of materials based on magnesium have been studied by many researchers in vitro; however, evaluations addressing the impact of the material in vivo still need to be improved. Several animal models, including rats, rabbits, dogs, and pigs, have been explored to assess the potential of magnesium-based materials. Moreover, strategies such as alloying and coating have been identified to enhance the degradation rate of magnesium-based materials in vivo to transform these challenges into opportunities. This review aims to explore the utilization of Mg implants across various biomedical applications within cellular (in vitro) and animal (in vivo) models.

## 1. Introduction

Since the 2000s, magnesium (Mg) and its alloys have attracted a lot of attention as potential materials for use in biomedical research [1]. High medical standards have been achieved by permanent implants; however, there are still several unresolved problems [2]. Conversely, bioresorbable magnesium-based implants have special properties that make them ideal for specific applications [3,4]. Their main advantage lies in their ability to degrade within a biological environment over time. Consequently, after fulfilling their role in supporting tissue healing and remodeling, there is no need for a secondary surgery for implant removal, thereby mitigating risks associated with general anesthesia and surgical and follow-up procedures [5,6]. Depending on the application scenario, many advantages can be observed. In orthopedic applications, magnesium alloys exhibit mechanical characteristics similar to cortical bone, avoiding the stress-shielding effects observed with more rigid titanium (Ti) alloys or stainless-steel implants [7,8,9]. Moreover, magnesium implants show promise in cardiovascular applications, such as coronary stents, as they are less thrombogenic than permanent implant materials, and their biodegradability enables restoration of vascular contractility in stented segments and preserves the growth adaptability of treated arteries, which is beneficial for pediatric patients [10,11]. It is crucial to remember that, in order to reduce the risk of thrombosis, Magmaris scaffold use usually requires temporary dual antiplatelet therapy (DAPT) right after implantation. Moreover, even though the resorbable nature of these scaffolds offers several benefits, there are still worries about late and very-late stent thromboses. To reduce these risks, it is crucial to carefully choose patients and treat them after implantation according to current research and user group guidelines [12].

Magnesium-based implants have many benefits in clinics, but one of the biggest obstacles to their application is the understanding of the fundamental processes occurring at the interface between the implant and tissue, or in other words, the host–biomaterial response [13,14]. It is still very challenging to create the best implants for different uses without a complete understanding of these mechanisms, considering material composition, manufacturing processes, surface modifications, and implant design, all of which have a significant impact on degrading behavior and the success of the implant [15,16]. Furthermore, the impact of releasing magnesium ions and the formation of other degradation byproducts on the biocompatibility of the biomaterial remains unclear, concerning the cells directly interacting with it in vitro [17]. On the other hand, it is critical to assess the safety and efficacy of magnesium-based implants using preclinical research in both small and large animal models before looking into their impact in clinics [7,18]. Animal research involving Mg-based implants offers valuable insights for preclinical assessment and paves the way for subsequent clinical trials. Thus far, numerous studies have studied the in vivo biocompatibility, degradability, and osteogenic potential of Mg-based implants [7,16,17]. When choosing an animal model for research, several factors are taken into account, including the animals’ availability, their pathophysiological traits comparable to human characteristics, the size and quantity of implants, the length of the observation period, the viability of surgery, and the difficulty of measuring the results [19,20,21]. It is critical to note that these considerations are not only aimed at achieving scientific accuracy, but also at adhering to the concepts of the 3R (Replacement, Reduction, Refinement). By following the 3R guidelines, we can make sure that animal studies are carried out cautiously, minimizing the waste of any resources while increasing the validity and reliability of results [22]. We aim in this comprehensive scientific review article to provide some invaluable insights that can pave the way for future preclinical in vivo investigations by first providing an in-depth exploration of the host–biomaterial response and its impact on implant success, and then analyzing research studies conducted both in vitro and in vivo, evaluating their study design, selection of cell and/or animal model, selection of magnesium materials, and evaluation techniques.

## 2. Mechanisms of Host-Biomaterial Interaction

The significance of biomaterials in medicine and the substantial growth in biomaterial science over recent decades is most evident in their global sales, projected to be USD 194.83 billion in 2024. This figure is predicted to rise at a compound annual growth rate (CAGR) of 14.8% from 2024 to 2032, at which point it is predicted to reach USD 587.08 billion [23,24]. Biomaterials encompass a broad range of materials and substances, including not only traditional implantable devices, but also drug and gene delivery systems, cell therapy substrates, bioreactors, biodegradable scaffolds incorporating growth factors and living cells for tissue regeneration, and micro- and nanoparticles for therapeutic and diagnostic applications [25,26,27].

Since the application of biomaterials into the human body, there have been ongoing concerns about their safety and effectiveness, prompting the exploration of clinical and preclinical evaluation methods [28]. The term ‘biocompatibility’ is defined as the ‘ability of a material to perform with an appropriate host response in a specific application’ [29,30]. As indicated, biocompatibility is context-specific, meaning it is defined within the framework of a particular application of biomaterials in the body, and not as a general property of the material itself [31]. The safety and successful clinical performance of a biomedical device are determined by the nature of the interaction between the material and recipient tissues, as well as the duration of this interaction [31,32,33].

A key and fundamental requirement for biomaterials is that they must not induce any harm or adverse effects on the host, either locally or systemically [29]. For instance, they should not exhibit cytotoxic, carcinogenic, or genotoxic properties, and should not induce reproductive toxicity [29,31,34]. Beyond the obligation of avoiding harm, biomaterials are designed to serve a beneficial function in the host, with the specific nature of this function varying according to the application [27].

Biomaterials implanted in the skeletal system, as an example, are expected to provide a specific response crucial for clinical functioning. This response involves the apposition of osseous tissue at the interface between the device and the surrounding bone, intending to avoid the interposition of soft tissue [35,36]. Furthermore, an ideal scenario involves rapid bone incorporation, ideally lasting throughout the entire life of the patient [37,38]. 

The first generation of biomaterials extensively utilized and researched in the field of medicine comprised long-term implantable devices, including artificial joints and metallic devices for osteosynthesis [39,40]. These materials were designed to be minimally biologically and chemically reactive, aiming to avoid interference with the natural healing processes of living tissues. For instance, materials such as titanium, cobalt-chromium, silicon, oxide ceramics, etc., were chosen for their resistance to corrosion and wear, as the release of ions, debris, or monomers could potentially disrupt tissue homeostasis [41,42]. 

In recent years, there has been a significant increase in expectations regarding the performance of biomaterials, with a focus now on devices cautiously interacting with tissues rather than being passively overlooked. Novel biomaterials seek to encourage active tissue engagement in generating a response that is not just compatible with but is also supportive of the desired outcome [27,37]. The potential requirements of biomaterials encompass different properties such as bioactivity, inductivity, drug elution, and biodegradability, in addition to the usual safety requirements [43,44]. 

For instance, a biodegradable device, such as a scaffold for tissue engineering, is not based on the principles of chemical and biological inertness. Instead, it is anticipated to interact with the body’s components, gradually degrading at a rapid rate to the growth of new tissue in its present form [45,46,47]. Throughout this process, it should release non-toxic and non-irritating by-products, maintaining a rate that prompts an appropriate local and systemic response. Importantly, this interaction is expected to occur only when its mechanical function is no longer necessary [16]. 

**Figure 1:**
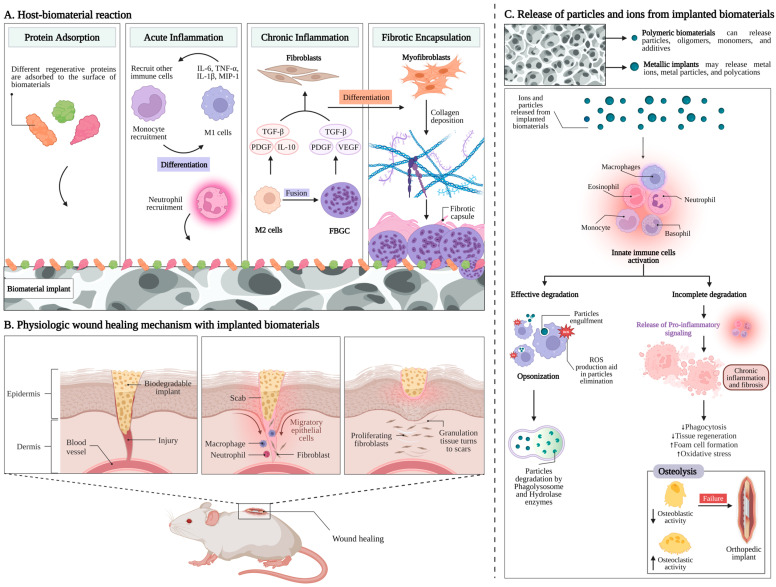
Host responses to implanted biomaterials. (**A**) Primary issues linked with host reactions after the implantation of biomaterials [48]. (**B**) The stages of wound healing in the presence of implanted biomaterial involve a dynamic interaction between the extracellular matrix and different cell types, including endothelial cells, platelets, fibroblasts, keratinocytes, and macrophages [67]. (**C**) Release of particles and ions from the implanted biomaterial and the subsequent degradation process [28,68].

The primary consequences linked to host responses post-biomaterial implantation are explained in detail in Figure 1A. The relationship between biomaterial surfaces and the response of the body focuses particularly on blood coagulation and complement activation [48,49]. Upon implantation, various blood proteins rapidly and non-specifically adhere to biomaterial surfaces, triggering inflammatory responses [50,51,52]. The coagulation cascade, initiated by factors like Hageman factor (FXII) and tissue factor (TF), is influenced by biomaterial surface properties, leading to thrombin generation and subsequent clot formation [53,54,55,56]. However, contact activation alone is insufficient, requiring platelet adhesion and leukocyte presence [57,58]. Components and products of the coagulation system, like fibrinogen and bradykinin, further modulate inflammation, affecting leukocyte activation and vascular permeability [59]. Additionally, the complement system plays a crucial role, and is predominantly activated through the alternative pathway upon biomaterial contact. Surface properties influence complement activation, amplifying inflammatory responses [60,61,62,63]. Anaphylatoxins released during complement activation attract leukocytes and promote vascular permeability [64]. Furthermore, complement activation can trigger platelet activation, contributing to coagulation [65,66]. This interplay between coagulation, complement, and platelet activation may provide insights into controlling subsequent inflammatory events. 

Following injury upon implantation, neutrophils are the primary leukocytes that migrate to the wound site, accompanied by exudation of fluid due to increased blood vessel permeability, which is a crucial characteristic of acute inflammation (Figure 1A) [69]. Various chemoattractants, including complement factors and fibrinopeptides, direct the recruitment and accumulation of neutrophils to the implant site [70,71]. Neutrophils play a crucial role as the first responders to defend against invading pathogens [71]. Their activation, including the release of reactive oxygen species, is influenced by biomaterial surface properties [72]. However, neutrophils have short lifespans and disappear from the site of inflammation relatively quickly, and circulating monocytes are then attracted to the injury site, where they differentiate into M1 macrophages [73,74]. These macrophages contribute to inflammatory responses by secreting proinflammatory cytokines and chemokines, which further recruit leukocytes to the injury area [75,76]. Additionally, macrophages produce reactive oxygen (ROS) and nitrogen species (RNS), which can have both antimicrobial and tissue-damaging effects [72]. While leukocytes can engulf smaller microorganisms, implanted biomaterials are too large for phagocytosis, leading to frustrated phagocytosis and the release of harmful radicals and enzymes that can damage surrounding healthy tissue [75,76]. This damage can result in the necrosis of larger tissue segments, posing a threat to patients [77,78]. Unlike neutrophils, macrophages have longer lifespans and become the predominant cell type in both acute and chronic inflammation, as well as during wound healing and fibrotic responses [79].

Continuous stimulation of tissues after the implantation of biomaterials can lead to chronic inflammation [80,81]. Macrophages play a pivotal role in regulating this inflammation, possessing a range of receptors mediating cellular behaviors [82]. Integrins, particularly α4/β1, α5/β1, α6/β1, and αX/β2, bind to different ligands and contribute to macrophage activation [83]. Upon activation, macrophages secrete proinflammatory factors and may coalesce to form foreign body giant cells (FBGCs) (Figure 1A) [73,82]. IL-4 and IL-13 induce FBGC formation, with surface properties and adsorbed proteins on biomaterials influencing this process [84,85,86]. β1 and β2 integrins mediate initial monocyte adhesion and macrophage development [87,88]. Macrophage fusion into FBGCs may depend on adhesion density and migration motility, highlighting the complexity of this process [89,90]. 

The host response to implanted biomaterials typically ends with fibrous encapsulation or fibrosis, driven by the interaction between macrophages and fibroblasts (Figure 1A) [91]. In the later stages of healing, macrophages transform to an alternatively activated phenotype ‘M2’, releasing some factors like PDGF and TGF-β1 to stimulate fibroblasts and promote collagen synthesis and wound healing [82,92]. Subsequently, fibroblasts differentiate into myofibroblasts, assisting wound healing and contributing to scar formation [93,94,95]. The prolonged presence of myofibroblasts due to continuous stimulation can lead to excessive collagen production and extensive fibrosis [96]. Fibrous capsules formed around biomaterials aim to isolate implants from host tissues but can lead to failure in medical implants [97,98].

Addressing these challenges requires the development of solutions dependent on the understanding of the chemical, biochemical, physical, and physiological mechanisms activated during the specific interaction between a biomaterial and host tissues [99,100]. The subsequent section provides a brief overview of the mechanisms involved in the interaction between biomaterials and the host, particularly pertinent to applications in tissue regeneration [99,101].

### 2.1. Wound Healing 

Surgical procedures induce trauma, and the introduction of the biomaterials into the body plays a role in the subsequent biological response (Figure 1B). This prompts immediate hemorrhage as blood vessels are damaged, triggering defense mechanisms that result in the formation of a blood clot [102,103]. Activated platelets release granules containing essential growth factors and biochemical signals, initiating processes such as vasoconstriction and the coagulation cascade [103,104]. Prothrombin and fibrinogen transform into thrombin and fibrin, respectively, stabilizing the formed coagulum, and initiating an inflammatory phase [105,106]. Hypoxia, resulting from interrupted blood flow, along with chemotactic factors released by the participating cells, prompts endothelial and mesenchymal stem cells to migrate to the region [107,108]. The blood clot, populated by various cell types, progressively transforms into granulation tissue and is eventually eliminated through fibrinolysis [102].

Angiogenesis is initiated, resulting in the formation of new vessels that provide nutrition and oxygen to the regenerating areas [109]. During this phase, progenitors of fibroblasts or osteoblasts can migrate into the tissue, likely utilizing the fibrin/osteoid matrix as a scaffold. Upon differentiation into active osteoblasts or fibroblasts, they begin to deposit collagen and other extracellular matrix components [102,110]. 

All of the previously mentioned events are characterized by normal wound or bone healing, and it is reasonable to assume that they also occur after implant installation [102]. A primary requirement for implants is, therefore, not to adversely impact the reparative ability of tissues but to facilitate the accumulation of new cells at the material interface. For instance, certain materials, such as copper, release toxic ions that inhibit the wound-healing process [111,112]. The use of silver nanoparticles in dermatology is growing because of their beneficial effects on healing and their ability to treat and prevent subsequent bacterial infections [113,114]. Another consideration is that implants should not experience excessive micromotion during the callus formation phase. Studies have shown that implants subjected to interfacial micromotion exceeding 150 µm during the healing phase are likely to be integrated by fibrous tissues rather than bone, possibly due to the instability of the blood clot caused by movement [115,116].

However, factors beyond the typical wound healing process contribute to the outcome of implant encapsulation by the tissue. The specific mechanisms governing the interaction between the host and biomaterial, leading to the encapsulation of implants, encompass mechanical, physical, and chemical elements, partially mediated by the immune system [103,117]. Recently, it has been proposed that the encapsulation of materials in the bone, as an example, represents a distinctive manifestation of the “foreign body response”, since biomaterials are inherently foreign to the body [118,119]. Unlike the soft tissue encapsulation observed around foreign bodies, the connective tissue that surrounds and isolates these implants in bone is bone tissue. This hypothesis was based on the observation that peri-implant bone exhibits histological differences from original bone, being more condensed and less innervated and vascularized [120,121], which is suggested to be different from the connective tissue encapsulating foreign bodies in soft tissues. However, it is crucial to note that the type of remodeling or replacement tissue around bone implants can vary significantly depending on the type of implant and its placement. Within this perspective, processes associated with the immune response to acute inflammation are proposed to play a more fundamental role in the establishment and maintenance of the host–implant interface than previously hypothesized [30,122].

### 2.2. Chemical Composition of Biomaterials

In recent decades, biomaterials research has primarily focused on implants with minimal chemical reactivity to the body [112,123]. The preference was for materials that were highly resistant to corrosion, and strategies were employed to reduce the release of particles and ions in the challenging conditions of the physiological environment [41,124]. This approach was justified due to the recognition that the removal of ions and debris from implants could harm the surrounding tissues. Implants are constructed from elements and materials that are intrinsically foreign to the host, resulting in potential toxicity [16,39,41]. In more recent research, a notable paradigm shift has become evident: inert implants are now subjected to chemical treatments to enhance their biological activity. There is a growing interest in the use of resorbable materials and corrodible metals for biomedical applications, where controlled biodegradation could provide clinical benefits [125,126]. These materials are intended to release chemical substances and metal ions in a controlled manner within the body. Consequently, it becomes crucial to explore potential chemically driven mechanisms of interaction between tissues and biomaterials [127,128]. It can be hypothesized that virtually all materials, even those considered bioinert, may possess chemically reactive surfaces, or can release some chemically active derivatives in a biological environment [129].

Titanium (Ti) is considered to be chemically inert due to the rapid formation of a 2–30 nm thick layer of titanium oxide (TiO_2_) when exposed to an aqueous environment. This oxide layer acts as a passivation barrier, shielding the titanium surface and preventing further chemical reactions with the surrounding fluids [130,131]. Consequently, titanium becomes resistant to corrosion and remains chemically stable in the physiological environment. However, it has also been reported that TiO_2_ can interact directly with proteins and macromolecules. Challenges arise in the clinical use of titanium implants due to the surface chemistry of Ti. Therefore, modifications have been made to the chemistry of titanium implant surfaces to increase the bonding of tissue [132,133]. Various chemical techniques, such as anodization or electrochemical oxidation, have been employed to alter the TiO_2_ layer [134]. These methods allow for the controlled development of oxide layers on titanium, simultaneously altering surface chemistry with other parameters like oxide crystallinity, porosity, and nanostructure [135,136]. Implants with such surface modifications have demonstrated enhanced tissue-to-implant contact in vivo [137,138]. However, it remains difficult to identify the specific impact of surface chemistry in this interaction, e.g., that of nanoporosity or surface charge [139].

### 2.3. Release of Particles and Ions

Chemical interactions between biomaterials and the host occur not only at the material surfaces, but also involve chemical molecules that the materials may release into the body [140]. All types of materials used in constructing biomedical devices have the potential to release degradation products, but resorbable materials are designed to do so in a controlled manner [28,68]. Polymeric biomaterials can release particles, oligomers, monomers, and additives, while metallic implants may release metal ions, metal particles, and polycations. Similarly, ceramics [23,39,141] and bioactive glasses [142,143,144] have the capability to release particles or ions (Figure 1C). 

The biological response from the host, and consequently the success or failure of the device in the body, is influenced by various factors including the amount, physicochemical status, chemical composition, emission rate, and concentration of the released particles [145]. The degradation products of materials may accumulate locally or be distributed throughout the body and can exist in stable or reactive forms. Even stable particulate that accumulates can trigger a physical reaction at the site [140,146]. Adverse reactions associated with the release of chemical particles in the body may include excessive inflammation, tissue necrosis, hypersensitivity, and tissue accumulation, as well as local or systemic toxicity and carcinogenicity [147,148]. It is crucial to understand the mechanisms by which the body responds to foreign elements and how these elements can evade the body’s surveillance to cause adverse effects. This knowledge is essential for the development of effective biodegradable materials and permanent implants [122,149].

Particles can be released into the host environment from biomaterials, implant coatings (such as plasma-sprayed hydroxyapatite coatings), or resorbable materials [150]. Additionally, particles can be intentionally delivered, such as microparticles and nanoparticles used in drug delivery systems [150,151]. The size of these particles is a crucial factor in determining their effects on the body. Nanoparticles, located within the range of 1 to 100 nm, can diffuse through cell membranes, while particles ranging from 100 nm to 10 m can undergo phagocytosis. When particles measure between 10 and 100 µm, multinucleated giant cells, formed by the fusion of multiple macrophages, engage in phagocytosis. Larger particles, which cannot be phagocytized by cells, can cause a phenomenon known as frustrated phagocytosis [152,153]. This can cause damage and necrosis in the tissues, and ultimately affect the material as well [154].

Most metallic particles produced are nanoparticles, although fractures of small pieces from implants may release larger particles. Moreover, polymeric materials frequently emit microparticles. Nanoparticles, with a larger surface-to-volume ratio than microparticles, generally exhibit higher reactivity [155,156].

When interacting with particles, cells of the innate defense system are typically activated. Dendritic cells (DCs) play a crucial role in the immune system as a mediator between biomaterials, bridging the gap between innate and adaptive immunity [157]. Moreover, neutrophils and macrophages can recognize and engulf particles covered by defensive molecules (such as complement factors) absorbed on the surfaces, a process known as opsonization [158]. Once internalized within these cells, the particles encounter the acidic environment of phagolysosomes and enzymes like hydrolases, which facilitate their digestion. Additionally, these cells can generate reactive oxygen species (ROS) that react with the particles, breaking them down through oxidation (Figure 1C) [158,159].

If the particles are effectively degraded by these mechanisms, as is the case with biodegradable materials, the host’s defensive reaction is usually terminated without any lasting effects [160]. However, if the particles cannot be degraded, the cells initiate the release of pro-inflammatory signals, leading to chronic inflammation in the area [128]. In the long term, this can result in fibrosis, isolating the irritated area from the rest of the body (Figure 1C) [122,160].

Phagocytic cells containing particles can migrate to lymph nodes, which serve as the immune system’s filtering stations, where non-degradable particles may accumulate. When particles exceed the size that can be internalized by a single cell, multiple macrophages may fuse to form multinucleated giant cells and attempt to internalize larger particles [154,161]. These cells typically trigger defensive mechanisms and sustain local inflammation by secreting pro-inflammatory cytokines and chemokines [159,160]. 

Osteolysis is a significant and challenging consequence resulting from the presence of non-degradable and irritating particles, particularly for orthopedic devices [162]. Non-degradable particles, macrophages, and multinucleated giant cells release chemotactic cytokines that attract osteoclasts to the bone, often leading to the clinical failure of orthopedic implants [160]. However, it has been documented that non-resorbable particles made of metal, ceramic, or bone substitute materials can become entirely encapsulated by bone, which is a shield-off mechanism. This outcome appears to be based on the ability of specific materials to modulate the activation of macrophages to the M2 type rather than the M1 type [163,164]. M1 macrophages represent the “pro-inflammatory” phenotype, sustaining inflammation and producing cytotoxic products such as reactive nitrogen and oxygen species (RNS and ROS), while M2 macrophages exhibit an “anti-inflammatory” profile, releasing cytokines and factors to suppress inflammation and promote wound healing [122,154,163,164]. 

Accordingly, it is important to choose materials with the ability to suppress excessive inflammation [165]. Furthermore, efforts have been made to design biodegradable polymers that selectively activate the M2 phenotype of macrophages [166]. In order to drive primary human macrophage elongation and differentiation towards the anti-inflammatory, pro-healing M2 type, a previous study showed that the control of biomaterial geometry using melt electrowriting to create fibrous scaffolds with box-shaped pores and inter-fiber spacing can potentially enhance tissue regeneration [167]. This is the motivation behind the exploration of Mg-based metals as biodegradable materials, recognized for their immunomodulatory and anti-inflammatory capabilities. Studies have demonstrated that Mg materials do not impair inflammation; instead, they exert control over it [168,169].

Traditionally, metal ions in the body have been considered toxic; however, metal elements are essential for cellular functions, such as catalytic, structural, and signaling functions (Figure 1C) [170,171]. The necessary metal ions for bodily functions are categorized into essential metals such as calcium (Ca), magnesium (Mg), sodium (Na), and potassium (K), requiring an intake higher than 100mg/day for health, and essential trace metals such as iron (Fe), copper (Cu), cobalt (Co), zinc (Zn), molybdenum (Mo), and chromium (Cr), which are crucial for the body, but should be present in very low amounts [171,172,173,174]. Certain metals, typically heavy metals, are not essential and can cause significant harm when present in the body, requiring complete caution. Examples include mercury (Hg), cadmium (Cd), lead (Pb), arsenic (As), and aluminum (Al), as well as others [175]. The body has developed endogenous protection systems for metal detoxification and excretion; however, when metal concentrations exceed the buffering capacity of these systems, toxic effects may occur. The toxicity of metals is influenced by factors such as the type of metal, its concentration, the duration of exposure, the oxidation state of the metals, and the health status of the host [170,176].

The presence of non-essential metal ions often leads to the formation of complexes with DNA or proteins within cells, where they can gain access through nonspecific diffusion across cytosolic membranes. Subsequently, these macromolecules become inactivated, resulting in cell death or degeneration [177,178]. An example is observed with Pb, which inhibits the synthesis of the heme group and thus, the production of hemoglobin. Another example is Hg, which binds to sulfur-containing molecules in the brain, causing them to become inactive and causing neurological damage. Alternatively, foreign metal ions can deactivate proteins and enzymes by competing for binding sites with Ca^2+^, as observed in the cases of Cd or gadolinium (Gd) [170,175,176].

Another challenge posed by metal ions within cells is their reactivity, which leads them to undergo reduction and generate high levels of ROS and RNS [159]. These reactive species then oxidize organic macromolecules, causing cellular damage. Metal ions may have toxicity even at sub-lethal concentrations, causing hypersensitivity by acting as haptens [179,180]. Haptens are small molecules or ions that bind to endogenous proteins, inducing conformational changes that label these proteins as “non-self” in the eyes of the immune system [160]. Macrophages and dendritic cells can internalize the protein–metal complex and present it to lymphocytes, resulting in the production of antibodies and specific immune responses. Metal hypersensitivity is commonly observed with nickel (Ni), and has also been reported with Co and Ti [181,182].

The release of metal ions from biomaterials has traditionally been a concern due to their potential toxic effects [183]. However, there is a growing interest in new applications that utilize controlled metal release for therapeutic purposes [39]. Bulk materials like Mg, Zn, and Fe have been suggested for implants designed to gradually biodegrade [184,185]. Implant coatings with Mg, Ca, Sr, and Zn ions have been developed to produce positive effects upon release in the body [186]. Al has been employed as a vaccine adjuvant [187]. Vanadium (V) is currently being tested for cancer treatment [188]. Silver nanoparticles and ions are actively studied for their antibacterial properties [189,190]. Mg-Ti particles, corroded through galvanic coupling, are being tested to reduce tumor growth [191]. Gd has been employed in anticancer therapies and magnetic resonance contrast agents [192]. It is important to note that these examples represent just a portion of ongoing research in this field.

With the emergence of these innovative technologies, particularly the growing interest in biodegradable metals for human applications, it has become crucial to clarify the effects of prolonged and substantial exposure to body-friendly elements such as Mg, Ca, Zn, Sr, Mn, and Fe [185,193]. Additionally, attention must be given to alloying elements like Gd, Ag, yttrium (Y), cerium (Ce), and neodymium (Nd), which are introduced into alloys to customize mechanical properties, and subsequently released in the body during material degradation [194].

Non-degradable implants made of Ti are frequently utilized for their favorable mechanical properties. However, they pose the risk of harming the fibrous tissue of the grafts and necessitate removal in a subsequent surgery [16,111]. To address this issue, biodegradable polymeric materials have been suggested, offering the advantage of natural resorption over time. However, these implants may face challenges related to insufficient mechanical strength or the potential induction of adverse reactions in the surrounding tissue during degradation. Furthermore, there is a desire to explore alternative materials and enhance tissue engineering in this area. One of these alternatives is Mg, which will be discussed in this review.

### 2.4. Status of the Host

Clinical observations demonstrate that individual patients exhibit various susceptibilities to implant failure, and the host’s local and systemic conditions significantly influence their response to biomaterials [195,196]. The successful establishment of a functional interface between biomaterials and the host relies heavily on the host’s ability to effectively activate wound healing and defense mechanisms. This capacity may be weakened in individuals with local or systemic conditions that hinder wound healing and immune responses, as well as by factors such as sex and age [122,197,198,199].

Health issues and systemic conditions, such as smoking or excessive alcohol consumption, may negatively impact the healing process, resulting in an increase in the risk of implant failure. However, it is important to highlight that, apart from medical emergencies and active infections, there are almost no absolute contraindications to implant placement [200,201]. Conditions that have the potential to disrupt the functionality of oral implants, for example, include bone metabolic diseases like osteoporosis, type 2 diabetes, smoking (in a dose-dependent manner), excessive alcohol consumption, head-neck irradiation, and the use of specific medications (cyclosporine, glucocorticoids, antidepressant drugs, and bisphosphonates) [202,203].

Recent studies have highlighted the influence of immunological signals on the interaction between biomaterials and the host, and the potential role of genetically or acquired immunological disorders in biomaterial rejection [28,204]. Similarly, rheumatoid arthritis (RA), an autoimmune condition, has been demonstrated to have a significant impact on bone loss around implants and implant failure, both in the jaw and in joint replacement procedures. This is likely linked to the degeneration of inflammatory processes in bone. The specific impact of RA itself, or medications used for RA, in the context of bone loss, remains unclear [205,206,207].

This inspired researchers to explore the correlation between certain genetic polymorphisms and their interaction with implants. It was discovered that certain genetic defects in pro-inflammatory markers are directly associated with increased peri-implant bone destruction [208,209]. The growing frequency of observations indicating that individual patient characteristics, including genetic polymorphisms in molecules crucial to inflammation, the extracellular matrix, bone remodeling, and coagulation, etc., could pave the way for a new area of study in biomaterials science [210] that we can call ‘biomateriogenomics’.

## 3. Properties of Magnesium and Its Functions in the Human Body

### 3.1. Physicochemical Properties

Magnesium, with an atomic number of 12 and symbol Mg, falls under the category of alkaline earth metals in group 2. It ranks as the ninth most prevalent element in the universe and is the fourth most abundant element on Earth [211,212]. Additionally, magnesium is found at significant levels in seawater, alongside sodium and chlorine. Mg, displaying an oxidation state of 2+ due to its high reactivity, naturally forms compounds with other elements [171,213]. While pure magnesium can be artificially obtained, appearing as a solid with a lustrous grey appearance, its inherent vulnerability to corrosion is noteworthy, with a standard electrode potential in water measuring −2.37 V [213,214]. Nevertheless, in atmospheric conditions, magnesium rapidly develops a protective oxide layer (MgO) on its surface, mitigating reactivity and providing effective corrosion resistance [214,215]. Corrosion risks are also avoided in liquids such as high-pH water (>10.5) or stagnant water, promoting the formation of protective hydroxide films (Mg(OH)_2_). However, the presence of aggressive electrolytes like chlorine or fluid flows can readily remove the hydroxide film, making Mg susceptible to corrosion under such circumstances [214,215]. 

Mg exhibits interesting characteristics that have been used for specific human applications. Its high flammability, for instance, has led to its use in weapons, explosives, light bulbs, photography, and fireworks [216]. Additionally, Mg has proven advantageous in transportation and aerospace contexts due to its status as one of the lightest metals, coupled with an impressive strength-to-weight ratio. To cater to specific engineering requirements, pure Mg necessitates alloying with other metals to fine-tune its mechanical properties effectively [216,217]. Beyond its exceptional strength-to-weight ratio, Mg alloys boast favorable qualities such as good castability, ductility, vibration and shock absorption, heat, and electricity conductivity, as well as being non-magnetic and non-toxic, collectively rendering them highly compelling for various applications [212,215,216].

However, Mg alloys possess a higher susceptibility to corrosion compared to highly pure Mg, particularly due to the presence of alloying elements that precipitate in secondary phase structures and intermetallic particles [218,219,220]. These alloying elements typically possess a significantly higher electrochemical potential than Mg, thus causing galvanic corrosion within the alloys. Furthermore, impurities such as Ni, Cu, Fe, and Co are nearly unavoidable during Mg processing, causing galvanic corrosion [219,221]. Additionally, Al alloys remain more prevalent than Mg alloys for applications where lightweight is a desirable property [219,222].

### 3.2. Biological Properties

**Figure 2:**
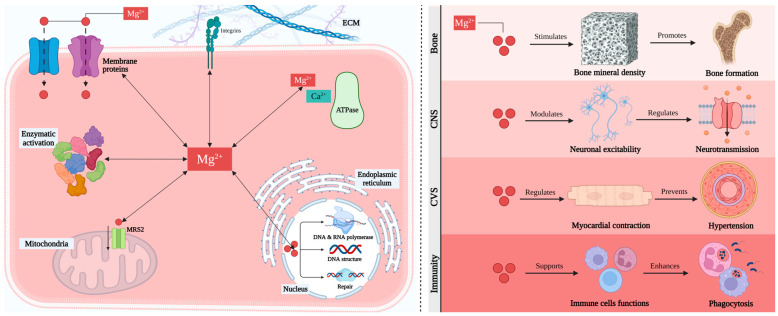
Fundamental aspects and functions of magnesium. **Left panel**: Intracellular magnesium distribution [236,237]. **Right panel**: Role of magnesium in different body systems [238].

Mg plays a foundational role in various cellular processes. Primarily, Mg is crucial for the chemistry of nucleic acids within the cells of several living organisms (Figure 2, left panel) [223,224]. The three-dimensional structures of RNA and DNA heavily rely on the presence of Mg ions. These ions bind to the negatively charged oxygen (O) and nitrogen (N) domains of these macromolecules, determining their functional integrity [225,226,227].

Additionally, Mg ions serve as coenzymes in over 600 enzymes and act as activators in another 200 enzymes [228]. Notably, Mg^2+^ plays a significant role in the activity of DNA and RNA polymerases, both of which have specific binding sites for Mg cations. The repair mechanisms of DNA are also extensively reliant on the availability of Mg. Consequently, it can be asserted that Mg is fundamental for maintaining genomic and genetic integrity [224,229,230].

Furthermore, Mg ions contribute to cell metabolism and glycolysis. Additionally, Mg^2+^ is a Ca^2+^ antagonist during cellular signaling and muscle contraction, indicating the importance of maintaining optimal concentrations of these ions [223,231]. 

Additionally, Mg ions are essential for cell interaction with the extracellular matrix through an integrin-mediated mechanism [232,233]. Consequently, they promote cell attachment to various substrates. The unique and fundamental chemistry of magnesium in the body is likely due to the dimension of its hydrated radius, which is a hundred times larger than that of other body cations (Na^+^, K^+^, and Ca^2+^). Despite its crucial role, Mg is often overlooked in medicine and has been referred to as the “forgotten cation” [234,235].

### 3.3. Effects of Magnesium on Health

Given the significant biological impact of Mg, it comes as no surprise that Mg plays a crucial role in the physiological functioning of various tissues, notably the brain, heart, and musculoskeletal systems (Figure 2, right panel) [238]. Inadequate intake of Mg has been linked to neurological disorders such as migraine, depression, and epilepsy [239]. The underlying reasons predominantly revolve around the modulation of synaptic signaling. Presently, Mg stands as an alternative therapy for migraine, with its efficacy subject to debate, but likely beneficial [240,241].

The availability of Mg may also influence cardiovascular health. Mg^2+^ ions play a crucial role in regulating myocardial contraction and electrical properties, as well as influencing vascular tone. Thus, supplementation with Mg could aid in the prevention of hypertension, myocardial infarction, and other cardiovascular diseases [242,243]. For instance, intravenous administration of Mg, typically in the form of MgSO_4_, is already widely accepted as a treatment for pre-eclampsia (gestational hypertension, high blood pressure during pregnancy), demonstrating clear beneficial effects despite only partially understood mechanisms [244,245].

Mg levels are crucial for the proper functioning of muscles. While calcium ions facilitate muscular contraction, magnesium ions compete with calcium to promote muscular relaxation. Consequently, Mg supplementation is commonly employed to relieve muscular cramps [212,223]. Additionally, given its importance in glycolysis, a correlation between low Mg intake and type 2 diabetes has been proposed as a contributing factor to this condition [224,246].

Magnesium also influences immunity and is recognized as an anti-inflammatory agent. This contributes to its role in the development of cardiovascular diseases, as a lack of magnesium impairs the inflammatory state in endothelial cells [247,248,249].

Ultimately, the availability of magnesium has a major impact on bone and cartilage health. Magnesium ions play a pivotal role in guiding the formation of hydroxyapatite (HA) crystals, thereby enhancing the solubility of calcium (Ca^2+^) and phosphate [PO_4_]^3−^ ions. In the absence of magnesium, HA crystals tend to be larger and more structured, rendering the inorganic matrix of bones brittle [250,251]. Furthermore, magnesium stimulates the proliferation of osteoblasts and promotes bone formation. Additionally, decreased levels of magnesium influence the expression of pro-inflammatory cytokines, which, in turn, trigger bone resorption [252,253].

The impact of magnesium ions on osteoclasts is multifaceted. On one hand, magnesium deficiency appears to enhance osteoclast proliferation, possibly due to the formation of a pro-inflammatory environment in low magnesium conditions. However, the attachment of osteoclasts to the bone matrix and their activation is partially facilitated by the presence of Mg^2+^ [254,255].

Additionally, magnesium seems to play a role in chondrogenesis. Prolonged magnesium deficiency has been found to inhibit the formation of chondrocyte columns and adversely affect the condition of articular cartilage in rats [256,257]. Conversely, elevated magnesium concentrations support the proliferation and activity of chondrocytes, suggesting magnesium as a potential tool for tissue engineering of cartilage [258].

Magnesium deficiency has been identified as a contributor to osteoporosis, acting through both direct and indirect pathways. Primarily, a decrease in magnesium ions in the bloodstream prompts the release of magnesium from bone reservations [259,260]. This reduction negatively impacts osteoblast proliferation and function, as well as the formation of hydroxyapatite crystals with diminished strength properties of the bone extracellular matrix (bECM). Furthermore, magnesium deficiency hampers the production and effectiveness of two critical hormones involved in calcium regulation: parathyroid hormone (PTH) and 1,25(OH)_2_-vitamin D [261]. 

Another indirect pathway through which a reduction in Mg levels contributes to the development of osteoporosis is by fostering a pro-inflammatory environment. Decreased Mg ions in the bloodstream stimulate the production of pro-inflammatory cytokines such as TNF, IL-1, and IL-6 in the bone marrow of rats [262,263]. These cytokines are known to enhance the activation of osteoclasts [264]. Furthermore, in the absence of sufficient Mg ions, oxidative stress is induced, and the effectiveness of antioxidants is diminished, both of which promote osteoclastogenesis and inhibit osteoblasts [265,266].

Moreover, as Mg ions regulate vasculature and endothelial function, low serum Mg levels could decrease vascularization in bone, rendering it more susceptible to osteoporosis [259,267]. Numerous studies in humans have identified a positive correlation between serum Mg levels and bone density in women. Magnesium deficiency is thus believed to contribute to osteoporosis. This assumption is supported by evidence showing that magnesium supplementation is beneficial for improving bone mineral density and preventing fractures in osteoporotic women [268,269]. On the other hand, excessive magnesium intake can cause diarrhea, nausea, and abdominal cramping. In severe cases, it may lead to dangerously high levels of magnesium in the blood, a condition known as hypermagnesemia. Symptoms of hypermagnesemia include low blood pressure, an irregular pulse, dyspnea, and in severe situations, cardiac arrest [270].

## 4. The Use of Magnesium in Biomaterials

**Figure 3:**
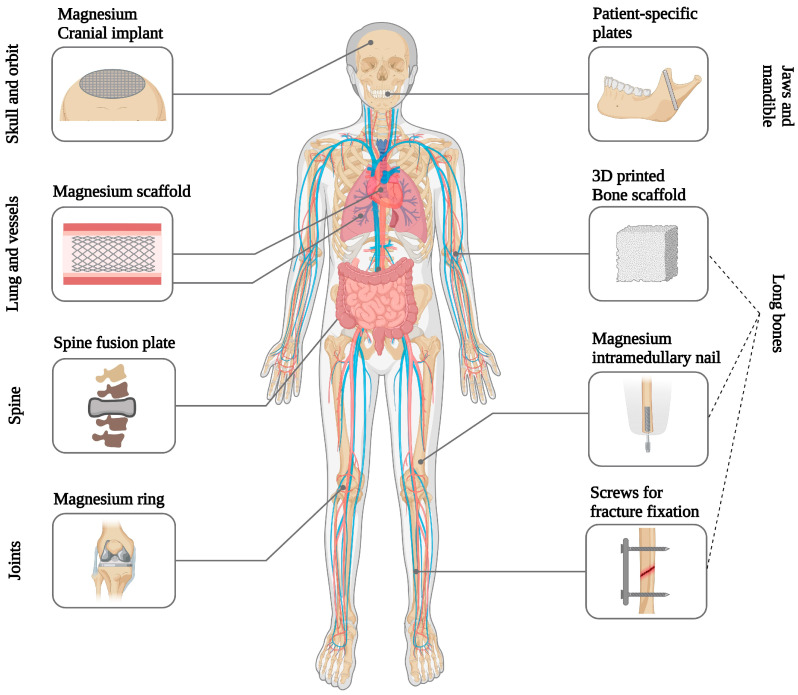
Magnesium-based implants are used in different clinical applications [5,13,274,281].

Since magnesium has been known to offer therapeutic effects since the 18th century, it is not unexpected that physicians explored the possibility of using magnesium-based biomaterials in the body not long after magnesium was first isolated as a metal [271,272,273,274]. The earliest reported application of magnesium as a biomaterial goes back to 1878, when Edward C Huse utilized magnesium wires as ligatures for blood vessels [13,275]. Subsequently, other clinicians explored innovative applications of magnesium in cardiovascular, orthopedic, and abdominal surgery, drawn by the metal’s ability to corrode in the body without causing toxic effects (Figure 3) [275]. Blood arteries, neurons, and intestinal tracts were proposed to be connected by magnesium tubes [276]. In bone surgery, magnesium screws, plates, and sheets were employed to resurface ankylotic joints, resulting in the successful restoration of joint motion and the fixation of fractures [277,278]. In general surgery, magnesium devices, such as arrows and wires, were used to halt bleeding in parenchymatous organs and the treatment of hemangioma cavernous (venous malformation) [279,280].

Despite the initial excitement among early clinical investigators, the utilization of magnesium materials experienced a rapid decline due to the formation of gas cavities in the tissue surrounding the corroding implants [17,282,283]. The primary challenge during that period was the inability to control the purity of magnesium, as impure magnesium underwent rapid and uncontrolled degradation, resulting in the intense evolution of hydrogen gas [284].

Today, owing to technological advancements in magnesium production, it is feasible to obtain ultra-high-purity magnesium or magnesium alloys with meticulously controlled mechanical and degradation properties [272,285]. Consequently, nearly two hundred years after their initial exploration, magnesium materials are once again being investigated with renewed interest in the field of medicine [275,286].

Magnesium-based biomaterials hold considerable appeal for diverse applications within the body. On one front, the inclusion of magnesium in permanent implants is suggested to improve material integration with the host tissue [13,283,287]. Conversely, investigations are underway for the use of implants composed entirely of magnesium or magnesium alloys in various applications, such as cardiovascular stents, osteosynthesis devices, and tissue engineering scaffolds for repairing bones, nerves, cartilage, tendons, ligaments, and other uses [18,193,283].

### 4.1. Magnesium-Doping of Permanent Implant Surfaces

Various modifications to the native oxide chemistry of titanium implants have been explored to enhance their clinical performance, particularly in challenging clinical scenarios [15,288]. One promising chemical modification involves the incorporation of Mg onto the titanium surfaces. Magnesium cations found in the TiO_2_ layer give proteins binding sites and can interact electrostatically with polyanionic proteins such as collagen, osteopontin, fibronectin, and vitronectin [289,290]. The presence of these proteins on the surface serves as a potential signal to attract osteoblast progenitor cells. These proteins are known to facilitate cell attachment to the extracellular matrix via transmembrane integrins. Once attached to the surface, the cells can differentiate into active osteoblasts and initiate the deposition of bone matrix directly onto the implant surface [291,292,293].

In theory, other cations could produce comparable outcomes, but research indicates that Mg^2+^ ions are the most effective in facilitating cell attachment to substrates [294,295]. Furthermore, Mg ions play a crucial role in the proper conformation of integrins, leading to enhanced integrin-mediated cell adhesion to the substrate with increased Mg ion availability [296,297].

The performance of titanium implants was previously investigated with incorporated magnesium in vivo [298,299]. Using micro-arc oxidation, titanium surfaces with magnesium incorporation were generated and significant enhancements in osseointegration were observed, despite minimal changes in topographical parameters [300,301]. The magnesium-modified implants demonstrated increased resistance to removal from bone and exhibited greater bone-to-implant contact [302,303]. Moreover, chemical analysis at the interfaces indicated a transfer of calcium, phosphorus, and magnesium ions between the implants and the bone, supporting the hypothesis of a biochemical bond formation between the magnesium-treated surfaces and the bone [304,305].

Moreover, alternative methods of incorporating magnesium into titanium surfaces have been previously explored, and these studies have shown superior performance of these implants in vivo compared to native titanium surfaces [306,307]. Furthermore, various experimental studies have highlighted another advantage of surfaces enhanced with magnesium: the accelerated establishment of osseointegration compared to titanium surfaces [298,308]. 

These findings support the concept that chemically modifying the surface of permanent metals with magnesium is a promising strategy for ensuring rapid and robust encapsulation of the device within bone tissue [17,309]. Given that magnesium is naturally present in the body and has a high tolerance limit, there are no foreseeable risks associated with its application on implant surfaces [287]. Therefore, such modifications could be preferred over those involving chemicals or drugs with unknown long-term effects.

### 4.2. Biodegradable Magnesium Implants

One of the most appealing medical properties of metallic magnesium is its ability to degrade in the physiological environment, releasing non-toxic byproducts. Therefore, biotechnological applications where magnesium shows the most promise are those where gradual material degradation over time is desirable [13,193,283]. A relevant example is the production of devices that can be absorbed when they are no longer needed. Temporary implants can help avoid complications associated with the prolonged presence of the implant in the body, as well as those related to implant removal [9,15,39]. Additionally, magnesium-based metals have an advantage over biodegradable polymers, possessing mechanical properties similar to those of bone and thus enabling them to withstand functional loads [13,285,310].

Other bulk materials exist as candidates for biodegradable metals, including Zn, Mn, and Fe [311,312]. However, Mg presents the lowest risk of toxicity among these elements. While the no observable adverse effect level of magnesium is estimated to be between 240 and 420 mg per day, the equivalent level of iron is 8–12 mg/day, and that of zinc is 8–11 mg/day [313]. Moreover, the release of magnesium products during implant degradation is suggested to positively influence bone healing, given the significant impact of Mg on metabolism [314].

The concept of utilizing magnesium-based metals for biomedical applications has been around for some time, but it has experienced renewed interest in the past decade. This is attributed to advancements in magnesium alloy production [315]. The initial alloying systems tested for medical applications were those readily available in the industry, such as the AZ systems, which primarily incorporate Al and Zn, and the WE systems, based on yttrium (Y) and rare earth elements (REE). Alloys containing Al were considered unsuitable as biomaterials due to concerns regarding the potential neurotoxicity of aluminum [316,317].

Interestingly, fewer health concerns were raised regarding Y and RE, the primary components of the WE systems, despite Y and certain other RE elements being associated with hepatotoxicity. Consequently, they underwent extensive in vivo studies. Preclinical testing demonstrated tissue compatibility, without signs of excessive inflammation or allergies, leading to its selection for clinical testing [5,318]. 

While magnesium (Mg) materials offer intriguing properties and significant advancements have been made in Mg research over the past 15 years, Mg-based implants have not yet become routine in clinical practice. This is primarily due to significant challenges, particularly related to magnesium degradation [9,283,319]. 

### 4.3. Challenges to the Advancement of Magnesium Implants

**Figure 4:**
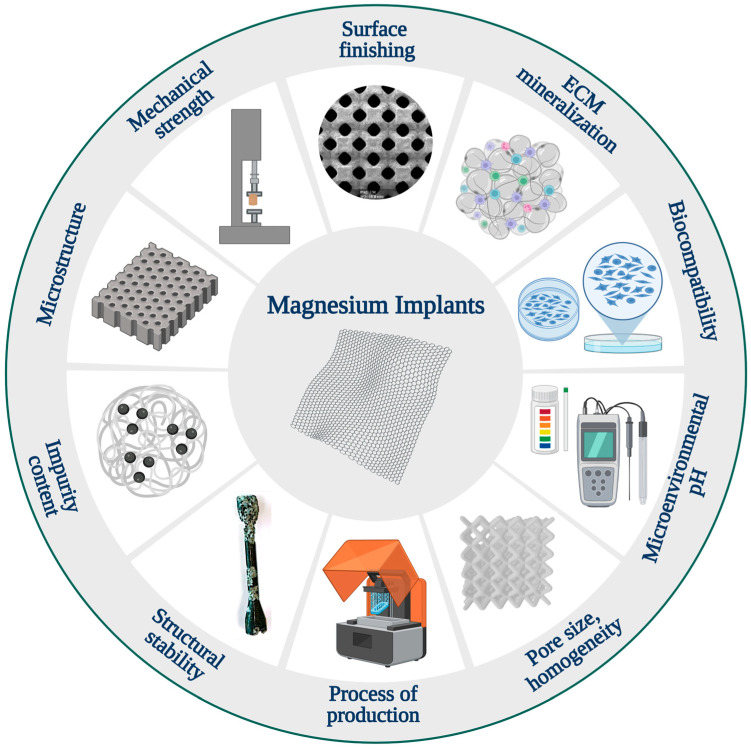
Some challenges involved in the advancement and development of magnesium implants [13].

There are several challenges in the study and development of magnesium implants that should be taken into consideration in the design process of these devices (Figure 4) [13]. Material degradation significantly impacts the successful performance of magnesium materials [3,13]. Rapid degradation can lead to excessive hydrogen (H_2_) evolution, potentially harming surrounding tissues [284,320]. The design and clinical application of magnesium implants must carefully take gas cavity formation into account. Excessive hydrogen gas evolution from these implants was found to cause massive subcutaneous emphysema, blood cell parameter imbalances, and decreased survival rates in rats [284]. Additionally, the nature and quantity of soluble and insoluble degradation products influence tissue response [13]. Furthermore, the timing and morphology of degradation affect the mechanical performance of the implanted material over time and tissue stimulation. From a metallurgical perspective, the introduction of alloying elements offers a method to mitigate corrosion while simultaneously altering the mechanical behavior of Mg [287,302,321]. 

Another challenge in the systematic study of magnesium alloys is that each processing step, from casting to the production of the final device, can result in significant alterations to the materials [216,322]. As a result, alloys with the same nominal composition but different manufacturing steps may exhibit markedly different degradation behaviors [13,219]. Factors such as microstructure, grain size, homogeneity, impurity content, and surface finishing all exert profound influences on material performance. Unfortunately, it remains unclear how each of these aspects affects material behavior [315].

Currently, there are no robust theoretical tools available for predicting the corrosion of magnesium alloys, which can only be assessed using experimental methods. In vitro corrosion tests are commonly used, but they fail to replicate the dynamic environment of living tissues accurately [219,323]. 

Mg^2+^ ions have varying effects on cellular responses in vitro depending on concentration, exposure duration, and cell differentiation state [324,325,326]. Concentrations of 2–10 mM enhance cell metabolism, proliferation, and early differentiation, but inhibit late differentiation and matrix mineralization [325,327,328,329]. Concentrations above 18 mM are toxic and reduce cell viability [330,331].

Mg^2+^ ions induce changes in microenvironmental pH, leading to alkalinity, which can disrupt cellular reactions [326]. Cells can recover from mild alkalinity by adjusting internal pH over time [326]. Severe alkalinity, however, affects cellular functions significantly, causing cell contraction, detachment, and reduced viability [332]. Severe alkalinity compromises human mesenchymal stem cell (MSC) renewal capability and growth, downregulating the proliferation rate [333]. Mild alkalinity up to pH 8.5 has no significant negative effect on osteoblast differentiation [333]. 

Many efforts have been directed toward the study of inflammatory and cellular responses to different biomaterials by using various in vitro and in vivo models, since biomaterial-induced chronic inflammation and fibrotic encapsulation are thought to be the primary causes leading to implant failure [323,334,335,336]. Efforts have been undertaken to develop in vitro models that closely mimic the physiological conditions of various tissues [337]. Here, we will discuss some in vitro models that are commonly used to test the biocompatibility of magnesium implants.

## 5. In Vitro Models for Studying Host Responses and Biocompatibility with Magnesium and Its Alloys

The biocompatibility of magnesium and its alloys has been assessed across various cell types, primarily fibroblast cell lines, primary cells, or cell lines originating from bone or vascular tissues, depending on the intended application [18,338,339]. However, different findings have been described due to various factors including variations in experimental protocols, parameters evaluated (such as cell adhesion, morphology, proliferation, and metabolic activity), and the utilization of diverse materials differing in composition, geometry, and manufacturing methods. Moreover, the outcomes of these studies often vary widely, and their ability to accurately predict corrosion rates in clinical settings remains limited [337,340]. In vitro tests have also been reported to inadequately replicate the conditions present in living tissues, and are unable to predict the behavior of alloys observed in in vivo experiments [337]. 

Many studies have reported favorable biocompatibility of magnesium salts [341,342] and various magnesium alloys with bone cells [342,343,344,345]. It has been previously demonstrated that magnesium can enhance bone cell adhesion on biomaterials through integrin expression [297] and the MAP kinase pathway [346]. However, other findings suggest the cytotoxicity of magnesium and its alloys [347,348].

A biocompatibility assessment of magnesium-based implants for potential use in the cardiovascular system has been conducted [193,283]. No toxic effects were found on human endothelial cells and vascular smooth muscle cells when exposed to high concentrations of magnesium salts [349]. Similarly, good cell viability of endothelial cells was observed around MgCa alloys, although no colonization of the materials themselves was noted [350]. Additionally, investigations into the hemocompatibility of magnesium alloys have yielded inconsistent results depending on the specific alloy type [193,339,351,352]. Favorable biocompatibility of MgCa extracts was demonstrated with L929 fibroblasts [353,354] and murine dendritic cells [341]. Additionally, no evidence of induced DNA damage, chromosomal aberrations, or gene mutations was observed from extracts of magnesium phosphate bone cement [355,356].

Given the variability in experimental protocols and outcomes, a comprehensive systematic study was conducted into various binary magnesium alloys to offer consistent data across different materials and cell types. Binary Mg alloys containing 1% Al, Ag, In, Mn, Si, Sn, Y, Zn, or Zr were utilized with pure magnesium as the control, and they were evaluated for their mechanical properties and corrosion behavior, as well as their hemocompatibility and cytotoxicity [357]. Two types of murine fibroblasts (L929 and NIH3T3), murine preosteoblast MC3T3-E1, human endothelial cells ECV304, and rodent vascular smooth muscle cells (VSMC) were used for the cytotoxicity tests by indirect contact using medium extracts. The extracts from aluminum, tin, and zinc alloys did not impact fibroblast and osteoblast viability; however, extracts from pure magnesium were found to have a slight effect on all cell types except L929 cells [357]. Based on these findings, it was recommended to use Al and Y for magnesium alloy stent production, while Ca, Zn, Sn, Si, Mn, and Al were considered suitable for orthopedic implants [357].

As magnesium corrosion produces hydrogen and prevents cells from adhering to the implant material, most biocompatibility assessments have been conducted by indirect contact with degradation extracts, in accordance with accepted guidelines (ISO 10993-5:2009) [358,359]. However, this method has its drawbacks, as it may not incorporate all degradation products. Theoretically, magnesium corrosion can yield four types of products: [OH]- ions, evolved hydrogen, discharged particles, and the release of metal ions (magnesium and alloying elements) [357]. Hydrogen dissolves during the preparation of extracts, and any particles are usually eliminated through centrifugation or precipitation processes [360]. However, when the extracts are directly supplemented into the cells, there is a risk of osmotic shock damage due to the high ion content and pH, leading to an overestimation of cytotoxicity [275,344]. Pure magnesium extracts were shown to have good compatibility with various cell types [341,357], and a recent study has revealed a cytotoxic effect when cells directly interact with magnesium [361,362]. 

It is important to evaluable biodegradable metals not only using biocompatibility testing methods, but also through investigation of genetic regulation markers [363,364]. Gene markers that can be investigated to assess the biocompatibility of degradable metallic materials include matrix metalloproteinases, antioxidants, cytokines, ICAM-1, and VCAM-1 for cell adhesion, p53, and p21 for cell death, IL-1, IL-6, TNF-α for inflammatory response, and TGF and SMGF for cell proliferation [363].

## 6. In Vivo Models for Magnesium-Based Implants

**Figure 5:**
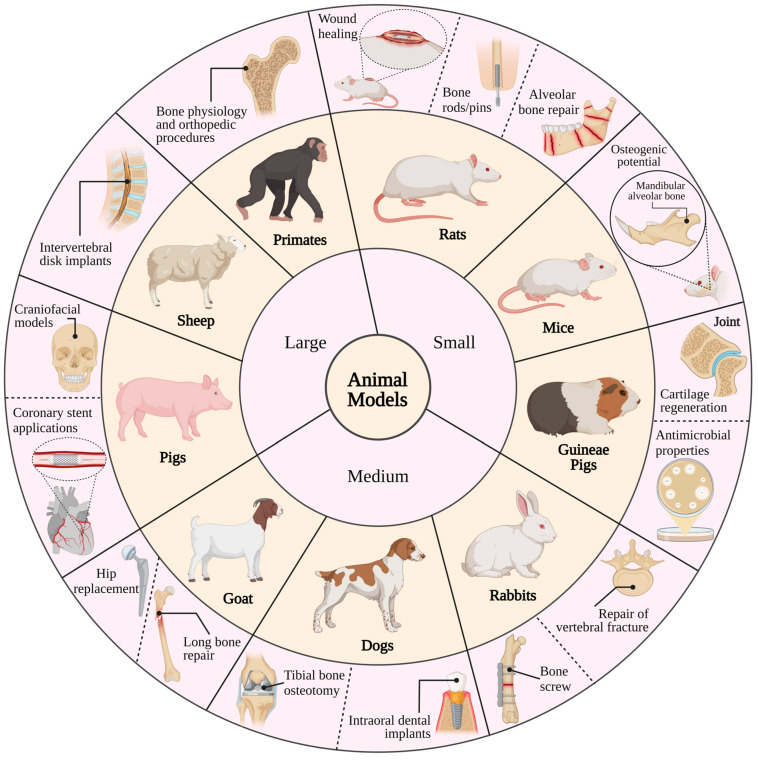
Common animal models used for the research of magnesium-based implants in biomedical applications [377].

Cellular or in vitro research cannot replace investigations using animal disease models [365]. For instance, assessing implant degradation, gas cavity formation, the local and systemic distribution of corrosion products and their impact on re-vascularization, and potential negative inflammatory responses are crucial for examining the long-term performance and safety concerns associated with biodegradable magnesium and its alloys [7,13,283]. This suggests that reliable insights into the biological, physical, and mechanical properties of magnesium may only be obtained through in vivo testing. To achieve the goal of introducing magnesium implants to the market, there is a need to standardize in vitro models that specifically address the biological effect of magnesium in a manner relevant to this type of material [366,367]. Simultaneously, it is crucial to elucidate the behavior of potential alloy candidates through well-designed in vivo studies that employ high-resolution techniques and integrate them to obtain comprehensive insights into magnesium degradation within the human body [368]. Animal studies involving Mg-based implants offer valuable insights for preclinical assessment and serve as a basis for subsequent clinical investigations (Figure 5) [325,369]. 

Following the selection of the implant material and design for the study, the material must be thoroughly investigated for various factors, including: (i) degradation rate, as degradation may be accelerated by increased bone turnover rate, (ii) physis, enhanced accumulation of particles within the physis, (iii) biocompatibility and immunological response alterations, and (iv) long-term assessment of any accumulated or potential (mildly) toxic particles or ions [370]. Several considerations guide the selection of appropriate animal models, including their availability, resemblance to human pathophysiology, implant size, quantity, observation duration, surgical feasibility, and data collection complexity (Figure 5) [21,325].

Developing biodegradable materials poses challenges not only due to differences between in vitro and in vivo conditions, but also due to heterogeneities among various animal models [371,372]. Previous in vivo studies have primarily aimed to explore the behavior of magnesium-based implants in non-fracture models, focusing on biodegradation and biocompatibility in general [373,374,375,376]. These studies have involved investigations into different types of magnesium materials, implant designs, animal species, and various methods for inducing fractures [373,374,375,376]. 

Another study aimed to compare the rare earth element (REE)-free Mg-based implant ZX00 in both small and large animal models [370]. The investigation focused on implant degradation, gas evolution, bone formation, and in-growth to demonstrate the feasibility of conducting biomaterials research related to bone formation in small animals, thus reducing the necessity for large-animal studies and associated expenses. Gas evolution was adequately observed in both small and large animal models without compromising bone formation or in-growth. Stress corrosion outside the bone in the surrounding tissue was observed mainly in the small animal model. Degradation rates were similar in both models and comparatively low, likely due to the high-purity Mg-Zn-Ca alloy used [370]. The study concluded that implant degradation in rats and sheep is comparable, suggesting the utility of transcortical implantation for investigating degradation rates and bone formation in a growing animal model [370]. 

Biodegradable Mg-based implants have gained attention due to their mechanical properties and potential for bone repair, especially with the introduction of MAGNEZIX^®^ [7,378]. However, concerns remain regarding implant degradation and mechanical failure [18]. While rats and rabbits are commonly used in Mg implant studies, larger species like sheep and goats offer advantages for evaluating implants in long weight-bearing bones [379]. Adequate consideration of anatomical sites, fixation methods, and evaluation techniques is crucial for reliable preclinical assessments [380]. 

Another study investigated the degradation behavior and osseointegration of WE43MEO(c) magnesium implants with and without plasma electrolytic oxidation (PEO) surface modification in miniature pigs [381]. PEO-modified implants KERMASORB(c) exhibited increased residual screw volume and bone-implant contact area at six months post-surgery. Surrounding bone density remained unaffected by surface modification. These findings suggest that PEO surface modification improved osseointegration and reduced the degradation rate within the initial six months, leading to increased bone growth overall [381].

In a recent systematic review of preclinical studies of Mg implants and fracture healing, rats and rabbits emerged as the most frequently utilized animal species [382]. Among the studies, 35% were performed using rats, and 30% used rabbits. Miniature pigs were used in 15% of the studies, and beagle dogs were used in 10% of the studies. Only one study utilized mice and goats as model species [382]. Notably, there was a specific preference for selecting female rats in studies [383]. Studies involving rats and rabbits tended to employ a higher number of animals compared to those involving dogs and pigs [384]. Nonetheless, the number of animals varied considerably, even within studies using the same species [382]. Similar variability was also evident in the observation periods across different experimental settings. Generally, a more extended follow-up period provides greater insights into the degradation process of magnesium-based materials. However, some long-term studies failed to furnish detailed results, such as the quantification of implant volume changes [385,386]. Among the studies included, 30% evaluated pure Mg, while 20% focused on Mg-Al-Zn-Mn alloy. Another 20% investigated Mg-Y-RE-Zr alloy, with an additional 10% evaluating Mg-Nd-Zn-Zr alloy. Furthermore, Mg-Ag, Mg-Ca-Zn, Mg-Ca-Mn-Zr, and Mg-Y-Zn-Zr-Ca alloys were each evaluated in one study [382]. Among the studies, 40% examined the impact of surface coating on biological response or implant degradation [382].

### 6.1. Selection Criteria and Assessment of Animal Models for Magnesium-Based Implants

When selecting an animal model, several key factors should be taken into account, including the level of intra- and inter-animal variation, as well as the availability of sensitive assessment methods for biological parameters (Figure 5) [387]. Additionally, the treatment conditions should closely mimic human clinical disease. In bone and joint models, specific considerations come into play, starting with identifying which sites and at what age the macro- and micro-structure of the model species best match human bone, especially considering the intended use of implants in cortical or cancellous bone [388]. Moreover, blood supplies supporting wound and bone healing can vary between species and by macroscopic structure, with cancellous bone typically having better vascularity than cortical bone [388]. Furthermore, repair responses may differ between skeletally immature and mature animals, with younger animals often exhibiting more rapid healing. Age-related changes can also be influenced by sexual dimorphism, particularly in rodents [389]. Large animal models may be necessary for implants that cannot be scaled down, such as those used in joint replacement procedures [390,391].

Accordingly, some key characteristics of frequently utilized animal species in bone implant material studies should be highlighted, offering insights into their relevance to human bone physiology and pathology [392]. The species include mice, rats, rabbits, guinea pigs, dogs, sheep, goats, and non-human primates (Figure 5). Rodents, including knockout and transgenic models, are widely used in implant studies for their biological relevance [393]. They are employed to assess osteoinductive and cartilage regenerative potential, as well as to model bone infection [394] and surgical approaches [395]. Although guinea pigs are not frequently utilized in bone implant research, specific strains, such as Dunkin Hartley guinea pigs, which exhibit spontaneous degenerative joint disease, are employed to assess cartilage regeneration and joint support implants. Male guinea pigs of the Dunkin Hartley strain develop histological lesions by three months of age, progressing within six months to lesions resembling those observed in human joints [396,397]. Rabbit hindlimbs are frequently employed in studies involving both cortical and cancellous bone implants, with the tibia being easily accessible due to minimal soft tissue interference, and the femur providing sufficient medullary space for internal fixation investigations [398,399]. Apart from bone studies, rabbits are also utilized in modeling vertebral fracture repair and evaluating methods to control implant-related pathogens [398,400]. Rapid cortical bone remodeling in young rabbits may not fully represent healing responses in adult humans, and their relatively fatty bone marrow is not ideal for autogenous bone grafts [401]. Dogs serve as common models in dental studies and offer a valuable model for studying peri-implantitis, characterized by tissue inflammation adjacent to dental implants, owing to their susceptibility to biofilm buildup and spontaneous periodontitis [402]. Large-breed dogs are particularly useful as they can often adapt human-sized implants, and their large synovial joints, like the stifle, allow for monitoring via arthroscopy [395]. Moreover, the proximal humerus of dogs provides enough material for autogenous bone grafts [387]. Additionally, dogs are well-suited for studies examining the impact of rehabilitation activities on implant functionality [399]. 

Both conventional pigs and minipigs are employed in craniomaxillary facial surgery models to assess osteogenic materials for bone repair [393]. These models are also utilized for extra- and intra-oral surgical procedures involving dental implants [395], interventions for femoral head osteonecrosis, and the treatment of cartilage and bone fractures [402]. Additionally, in skeletally mature conventional pigs, the articular cartilage is thick enough (approximately 1.5 mm) to create both full- and partial-thickness defects, which can be monitored using arthroscopy [399]. 

The utilization of small ruminant orthopedic models, including sheep and goat models, rose from approximately 5% of bone studies in the 1980s to 11–15% in the 2000s. These animals are employed to simulate defect repair in long bones, assess bone filler materials for cranial defects, evaluate fracture repair devices, study tissue response to wear debris, explore extraoral surgical techniques, and investigate intervertebral disk replacements [394,395]. Additionally, the relatively vertical alignment of the cervical vertebrae in small ruminants makes them suitable for modeling some axial compression and rotation forces similar to those experienced by the human spine [387].

### 6.2. Animal Models for Magnesium-Based Materials

Inadequate study design or improper model selection often results in many animal studies having little value or being redundant from a translational perspective. Collaboration between material scientists, biologists, statisticians, and physicians is essential to improve model selection, methodological quality, and translational efficacy for the development of biodegradable magnesium implants. Therefore, the upcoming section will delve into the animal models employed for investigating Mg-based materials.

#### 6.2.1. In Orthopedics

**Mice models:**

The Mg alloy ‘Mg2Ag’, containing 2% silver and known to exhibit promising mechanical properties, degradation rate, and biocompatibility in vitro [403], was implanted in intramedullary nails into mice, both with and without femoral shaft fractures [371]. A faster degradation rate was observed in vivo compared to in vitro, yet without any observed health abnormalities stemming from degradation [371]. Additionally, the Mg2Ag alloy demonstrated an inhibitory effect on osteoclast function both in vitro and in vivo, and it promoted bone formation during bone remodeling while reducing bone resorption [371]. Another study was conducted using an immunocompromised mouse model to explore the long-term biological effects of Mg alloy in vivo and its impact on human bone marrow stromal cells [404]. Pure Mg or Mg alloy ‘AZ31’ was implanted into collagen sponge scaffolds seeded with human bone marrow stromal cells and then subcutaneously implanted in mice [404]. The degradation and biological effects of the implants revealed that pure magnesium degraded more rapidly than AZ31, yet both exhibited good biocompatibility after eight weeks. Immunohistochemistry analysis demonstrated the expression of matrix protein 1 and osteopontin around the implants, along with the presence of a thin mineral layer surrounding the implants [404].

**Rat models:**

Rats have been extensively utilized to explore the efficacy of pure Mg implants. In a study by Hamushan et al., a distraction osteogenesis model in Sprague Dawley rats was employed to assess the impact and mechanism of high-purity Mg pins on osteogenesis [405]. The results revealed that Mg implantation significantly enhanced both the quantity and quality of healed bone tissue, leading to a faster consolidation process during repair [405]. Moreover, owing to its high purity, Mg implants exhibited stable degradability throughout the experiments. Through RNA sequencing analysis and other techniques, the authors identified that Mg promotes osteogenesis by modulating patched 1 protein, thereby activating Hedgehog-alternative Wnt signaling pathways [405]. In another study, a 99.99%-pure Mg rod was employed in a Sprague Dawley rat non-fractured femur model [406]. A notable increase in Mg concentration was detected two weeks post-surgery in the cortical bone and bone-periosteum junction of the femur implanted with Mg, indicating significantly elevated Mg levels in these areas and substantial new bone formation in the cortical bone [406]. Immunofluorescence staining revealed a high concentration of calcitonin gene-related peptide in the peripheral cortical bone implanted with Mg. Interestingly, the removal of the periosteum before implantation led to a significant reduction in new bone formation in this area [406]. It was proposed that Mg stimulates the secretion of calcitonin gene-related peptides by axons on the bone surface, thereby promoting new bone formation, a process hindered by periosteum removal [406].

Periprosthetic infections pose significant challenges in orthopedics, often demanding prolonged antimicrobial therapy, implant removal, and surgical revision. To assess the antibacterial efficacy of pure Mg in vivo, pure Mg was implanted in intramedullary nails in 5-month-old Sprague Dawley rats [407]. The Mg implantation notably mitigated bone destruction caused by infection and effectively protected bone and adjacent tissues from methicillin-resistant *S. aureus* infection [407]. Additionally, the Mg implant group exhibited lower total porosity and fewer pores, indicating that pure Mg intramedullary nails could inhibit bone destruction due to infection and stimulate bone formation around the implant [407]. The osteogenic potential of Mg on mandibular alveolar bone was investigated in a previous study, where pure Mg was implanted into rat mandibular incisor extraction sockets [408]. Mg concentration around the alveolar bone was significantly higher compared to the control group two weeks post-surgery, indicating a beneficial effect on cortical and trabecular bone repair. However, this effect was not sustained at six weeks post-operation [408]. Enhanced angiogenesis was also reported following Mg implantation in the second week after surgery, along with the presence of gas bubbles resulting from Mg degradation in the sockets [408]. In a previous study, the biodegradability of Mg-Ca-strontium (Sr) alloy and its impact on adjacent tissues was assessed, where Mg-1.0wt.% Ca-0.5wt.% Sr alloy pins were implanted into rat tibiae [409]. The implants were well tolerated, with only mild swelling observed within 4 days post-surgery and no signs of infection [409]. New bone formation replaced the degrading implant, resulting in excellent bone repair in 6 weeks. Despite reported local gas accumulation, no microfractures were observed in the bone [409].

**Rabbit models:**

A bone screw was previously designed for fixing bone fractures in the distal femur of rabbits [410]. These screws were crafted using high-purity magnesium (Hp-Mg) materials that had undergone a prior rolling process. Due to the mechanical stresses experienced at the implantation site, the corrosion rate exhibited a linear correlation with time, persisting up to 24 weeks post-operation. The Hp-Mg screws demonstrated excellent degradation and superior osteogenic performance, with irregular woven bone formations in comparison with a group utilizing poly-L-lactic acid, and no discernible biosafety concerns were reported [410]. In another study, the bone marrow environment was explored within fractured rabbit ulnae treated with Mg-based plates and screws [411]. Micro-CT analysis of rabbit ulna samples revealed the presence of radiopaque mineralized tissues within the medullary cavity at 8 weeks postoperatively, while extensive bone remodeling and increased mineralized deposition were observed at 16 weeks post-surgery [411]. It was proposed that the osteogenic effects of Mg are facilitated through the activation of the canonical Wnt signaling pathway [411]. Furthermore, Mg-based interference screws were found to exhibit good repair capabilities in a rabbit model of anterior cruciate ligament reconstruction [411].

The therapeutic potential of Mg-Cu alloys with different Cu content was investigated in treating osteomyelitis in rabbits [412]. In vitro studies showed that the Mg0.25Cu alloy exhibited significant antibacterial effects. Mg0.25Cu alloy was implanted into the model, revealing favorable biocompatibility and effectively suppressing bone infection, with minimal inflammatory response around the implant site and scarce inflammatory cells within the tibial marrow cavity [412]. Moreover, bone defects induced by infection were repaired, and regeneration of thin cortical bone was observed [412]. In another study, a novel coating for AZ31 Mg alloy surfaces was developed using polydopamine (PDA)-mediated assembly of hydroxyapatite (HA) nanoparticles, with the addition of bone morphogenetic protein-2 (BMP-2). This coating enhanced the biocompatibility and corrosion resistance of the Mg-based implants while facilitating the sustained release of BMP-2 in vitro [413]. Rabbit models with critical-sized femoral defects were implanted with PDA/HA-coated AZ31 and PDA/HA/BMP-2-coated AZ31, and no abnormal behavior or signs of wound infection were reported [413]. Histological analysis revealed the absence of polymorphonuclear cells in all groups, indicating that neither the coating nor the exposed AZ31 after coating degradation exhibited adverse effects on the surrounding tissues. Enhanced bone repair was observed in the PDA/HA/BMP-2 group compared to the PDA/HA group, with smaller empty cavities observed around the new bone and implants [413].

**Other animal models:**

An absorbable Mg-Zn-Ca alloy (MgZnCa; <0.5 wt% Zn and <0.5 wt% Ca; ZX00) was recently developed for pediatric use and its degradation and bone formation properties were assessed in both small rodent and large ovine models [370]. The alloy was surgically implanted into the femurs of Sprague Dawley rats and the right proximal tibiae of 1-month-old female lambs. Gas release was observed post-surgery in both animal models; however, there was no notable inconsistency in the degradation rate of the implants between the two models. Moreover, osseointegration was evident based on micro-CT and histological assessments in both models [370].

In another study, the performance of anodized WE43 magnesium alloy (comprising magnesium, yttrium, rare earth elements, and zirconium; Elektron SynerMag^®^), monolithic WE43 magnesium alloy, and poly-L-lactic acid implants were evaluated in 1-year-old beagle dogs [414]. Tibial bone osteotomy was conducted in the dogs, followed by fixation using screws made from the test materials. The anodized WE43 and monolithic WE43 groups exhibited superior resistance to loosening and breakage compared to the poly-L-lactic acid group at 4 and 12 weeks post-surgery. Bone resorption and gas formation were reported around the monolithic WE43 implants, while the anodized WE43 group displayed minimal bone resorption [414].

The Mg alloy WE43 was utilized as an implant in the frontal bone of adult miniature pigs [415]. Half of the Mg implants experienced plasma electrolytic coating. No complications associated with the implants were observed. However, one-week post-surgery, subcutaneous gas pocket formation was observed around uncoated Mg implants due to implant degradation, which was absent in the coated Mg implant group. Moreover, after 12 and 24 weeks post-surgery, the coated implanted group exhibited superior bone formation and no signs of inflammatory cells or increased osteoclast numbers around the implants compared to the uncoated implant group [415].

#### 6.2.2. In Cardiology

Biodegradable Mg alloys hold promise as materials for cardiovascular stents (CVS), offering potential solutions to long-term clinical issues associated with current CVS, including in-stent restenosis and late stent thrombosis [10,416]. Stainless steel and cobalt-chromium alloys are commonly used for permanent stents, effectively preventing arterial restenosis after dilation [417]. However, their prolonged presence often leads to late in-stent restenosis due to arterial wall irritation, which is problematic in pediatric surgery where non-resorbable implants lack growth adaptability [417]. This is where magnesium alloy stents offer advantages. They support artery patency during remodeling, while their complete degradability eliminates post-implantation irritation, potentially restoring vasomotor function [418]. Moreover, magnesium has been suggested to possess anti-thrombogenic and antiarrhythmic properties, further enhancing its appeal for stent applications [10]. The viability and biocompatibility of magnesium alloys have been confirmed in cardiovascular stents through both animal [10,419,420,421,422,423,424] and clinical [425,426,427,428,429,430] investigations. 

In 2003, 20 prototype coronary stents composed of AE21 Mg alloy were implanted into the coronary arteries of 11 domestic pigs [419]. Results revealed complete coverage of the AE21 Mg alloy stent by vascular intima in the early implantation stages, with no evidence of thrombus or inflammation. However, between 10 to 35 days post-implantation, arterial lumen diameter reduction of approximately 40% occurred due to significant intimal hyperplasia, and faster-than-expected degradation rates of AE21 Mg alloys in vivo were reported [419]. In another study, WE43 Mg alloy (Lekton Magic^®^) stents were implanted in pig coronary arteries [431]. Endothelialization of vascular stents was observed within six days post-implantation, with suppression of smooth muscle cell proliferation. Moreover, histological analysis revealed degradation behavior of the implanted WE43 Mg alloy stent at 35 days post-implantation, with an anticipated complete degradation time of 98 days [431]. JDBM (Mg-Nd-Zn-Zr) stents were implanted previously into the abdominal artery of rabbits, demonstrating uniform degradation and support provision for up to six months. The peripheral vascular tissue showed modest inflammatory reactions, deemed acceptable clinically [10]. The ZE21B (Mg-Zn-Y-Nd) alloy implanted in the porcine coronary artery showcased excellent mechanical and corrosion resistance properties, confirming its efficacy and safety as an absorbable stent [422]. In two other studies, absorbable metal stents (AMS) were implanted in the porcine coronary artery. No indications of persistent inflammation were observed post-implantation and the lumen area reached its minimum at 3 months due to adverse vascular remodeling [423]. AMS stents, when compared to stainless steel stents, exhibited biocompatibility and induced less neointimal formation, with no change observed in the lumen area [424]. In coronary or femoral arteries in dogs, AZ91 Mg alloy was implanted and 2–4 weeks after stent implantation, the intima hyperplasia was mild, but there was no distinct inflammatory reaction and initial thrombosis [420].

#### 6.2.3. In Other Medical Applications

A recent study investigated a novel biodegradable magnesium (Mg) skin staple as an alternative to traditional stainless steel staples commonly used in clinical practice [432]. In vitro, the Mg skin staple (ZK60 Mg alloy) demonstrated favorable mechanical properties and favorable cell viability, indicating no harmful effects on human keratinocytes (HaCaT) and L929 mouse fibroblast cells [432]. In vivo assessment in rabbits revealed no tissue irritation and comparable wound healing between Mg and stainless-steel staples after 1–3 weeks. Some Mg staples spontaneously dislodged within three weeks, offering potential benefits of reduced long-term retention [432]. 

In another study, high-purity Mg staples were used to test their biocompatibility in vitro using the human colon carcinoma LS174T cell line and in vivo using New Zealand rabbits [433]. The results suggested that the staples can facilitate wound healing post-colorectal tumor resection while inhibiting the recurrence of residual tumor cells both in vitro and in vivo. Over 7 weeks of implantation, the Mg staples facilitated gradual intestinal wound healing without adverse effects such as leakage or inflammation [433]. Additionally, the implanted Mg staples demonstrated inhibitory effects on the growth of colorectal tumor cells and their migration to normal organs, attributed to the increased concentration of Mg ions and released hydrogen [433]. These antitumor effects were corroborated by in vitro cell experiments, revealing that Mg induces apoptosis of tumor cells and hampers their growth and migration [433]. 

Another study aimed to assess the skin-sensitizing potential of corroding implant materials made from various magnesium alloys, titanium, and a degradable polymer [434]. No skin sensitization in a guinea pig model exposed to the magnesium alloys was reported. Erythema observed after patch removal in animals treated with solid test substances was attributed to mechanical irritation rather than allergenic skin reactions [434]. Minimal basophile cell counts were reported histologically, indicating no cutaneous basophile hypersensitivity induced by the test substances. Despite some instances of erythema observed in animals treated with magnesium alloys AZ91 and LAE442, histomorphological examination of allergenicity was significantly lower compared to the positive control group, indicating no skin-sensitizing potential [434]. To assess the effect of magnesium hydroxide on wound healing in rats, two models of skin wounds were created, and the healing process was evaluated by measuring wound length and area, conducting tensiometry experiments, and analyzing histological samples [435]. It has been shown that treatment with magnesium hydroxide significantly accelerated wound healing compared to the control group, with higher percentages of wound closure observed at various time points. Additionally, tissue strength, as indicated by stress and strain measurements, was increased in the magnesium hydroxide-treated group [435]. Histological analysis revealed accelerated wound healing and cell aggregation in the magnesium group compared to controls [435]. 

Gupta et al. fabricated magnesium-doped silk fibroin film that can be used for skin regeneration, with considerations for maintaining physiological magnesium levels to avoid immune activation or dysfunction [436]. Magnesium ions trapped in the film are gradually released into the wound, creating an acidic microenvironment that resists bacterial infections. In vivo studies have demonstrated accelerated wound contraction and re-epithelization, accompanied by an influx of and subsequent decrease in inflammatory cells, indicating the efficacy of Mg-doped film in promoting wound healing and keratinocyte migration [436]. A bio-multifunctional hydrogel, CCOD-MgO, was developed in another study using double cross-linking with MgO-catechol and Schiff’s base bonds, combining MgO with catechol-modified chitosan (CHI-C) and oxidized dextran (ODex), and hepatic hemorrhage ICR female mice were utilized to test this hydrogel [437]. This hydrogel exhibited strong tissue adhesion, effective self-repair, hemostatic properties, and minimal swelling. It has been demonstrated that CCOD-MgO protects wounds from infection and accelerates epidermal healing in full-thickness cutaneous defects and burn models, suggesting its potential as a promising therapeutic strategy for the clinical treatment of such injuries [437]. 

Many research studies have revealed the microbial inhibitory properties of Mg-based alloys, attributed to the release of OH^−^ and Mg^2+^ ions, which possess antibacterial effects [438]. Additionally, the degradation products of Mg-based alloys, Mg(OH)_2_, and MgO nanoparticles have demonstrated antibacterial properties against various bacteria [439,440,441,442]. Nano-MgO exerts its effect through an acid-base reaction with bacterial walls [439,441], while nano-Mg(OH)_2_ disrupts cell wall integrity upon bacterial adsorption [442]. Pure Mg has been shown to exhibit antibacterial effects against *Escherichia coli*, *Pseudomonas aeruginosa*, and *Streptococcus aureus*, comparable to fluoroquinolone antibiotics [443,444], and has shown therapeutic potential against osteomyelitis caused by *Streptococcus aureus* and methicillin-resistant *Streptococcus aureus* (MRSA) [407]. Furthermore, Mg-based alloys containing antibacterial elements like Ag, Cu, and Zn have demonstrated enhanced antibacterial activity compared to pure Mg [403,445,446,447].

## 7. Conclusions

The utilization of Mg and its alloys as biodegradable implants in medical applications shows great promise due to their specific beneficial properties. These include high specific strength, low density, elastic modulus, degradability, and good biocompatibility. Despite facing some challenges, such as a rapid corrosion rate and limitations in load-bearing applications which can lead to premature mechanical implant failure, Mg and its alloys are therefore very interesting candidates for further development. Therefore, understanding their mechanical and corrosion behavior, as well as strategies to enhance mechanical strength and corrosion resistance, is crucial. The degradation process and the safety of an implant can be predicted through in vitro degradation experiments and animal models. Based on the research findings highlighted in this review article, several crucial factors must be taken into account when developing in vivo experiments using magnesium implants. For the efficacy and safety of Mg-based implants, various small and large animal species should be considered following the 3R principles, along with other factors such as anatomical site and surgical techniques. Additionally, it is important to employ animal models that closely mimic clinical scenarios and select appropriate methodologies for evaluating bone and wound healing and implant degradation. It is essential to consider the practical and ethical challenges associated with conducting animal experiments, especially those involving complex surgical procedures. Considering the previously mentioned aspects during study design will enable researchers to formulate robust research plans with significant clinical relevance. This review aims to offer insights into selecting clinically relevant animal models, designing implants, and devising evaluation methodologies, thereby aiding in the planning of preclinical research with a translational outlook.

## Data Availability

All data presented in this review are available in the manuscript.
